# Impact on early trauma mortality of the adoption of the Updated European Guidelines on the management of bleeding

**DOI:** 10.1186/cc11057

**Published:** 2012-03-20

**Authors:** E Cingolani, G Nardi, G Ranaldi, C Siddi, S Rogante, A Ciarlone

**Affiliations:** 1 Azienda Ospedaliera San Camillo Forlanini, Roma, Italy; 2 S.Camillo Hospital, Roma, Italy

## Introduction

Post-traumatic bleeding is the leading cause of potentially preventable death among trauma patients. The Updated European Guidelines (UEG), published at the beginning of 2010, were aimed to provide an evidence-based multidisciplinary approach to improve the management of the critically injured bleeding trauma patients. The aim of this study is to evaluate the impact of the implementation of UEG recommendations on early hospital mortality for severe trauma in a high-flow trauma center.

## Methods

S. Camillo Hospital is a level 1 trauma center based in downtown Rome, with a catchment population of 2.5 million people. UEG recommendations were formally adopted and implemented since 1 April 2010. The pre-existing hospital guidelines were modified as follows: immediate pelvic ring closure for all unstable patients with a suspected pelvic fracture; early administration of plasma with a higher rate of plasma/blood units; early use of thromboelastometry to monitor bleeding patients; and early use of antifibrinolitics for all bleeding patients. Data on trauma admissions and early hospital (6 hours) mortality before (2009) and after the adoption of the UEG were collected using the hospital registry, and were subsequently analysed.

## Results

A total of 1,617 patients met the criteria for full trauma team activation (551 in 2009, 528 in 2010 and 538 during the first 11 months of 2011). There were no differences for gender, age, mechanism of injury and average ISS. In 2009 21 patients died within the first 6 hours versus 17 in 2010 and 12 in 2011; *P = *0.3, *P *for trend = 0.1 Hemorrhage was the most important cause of death within this time-span. All early trauma deaths occurred in the operating room or in the emergency room during the initial stabilization.

## Conclusion

This is a retrospective cohort study based on the data of the S. Camillo Hospital registry and the emergency department electronic shift. With the limitations of all retrospective studies, our data suggest that the implementation of the European Guidelines recommendations might contribute to a relevant reduction in early trauma mortality.
